# Inherent limitations of probabilistic models for protein-DNA binding specificity

**DOI:** 10.1371/journal.pcbi.1005638

**Published:** 2017-07-07

**Authors:** Shuxiang Ruan, Gary D. Stormo

**Affiliations:** Department of Genetics and The Edison Center for Genome Sciences and Systems Biology, Washington University School of Medicine, St. Louis, Missouri, United States of America; Ben-Gurion University of the Negev, ISRAEL

## Abstract

The specificities of transcription factors are most commonly represented with probabilistic models. These models provide a probability for each base occurring at each position within the binding site and the positions are assumed to contribute independently. The model is simple and intuitive and is the basis for many motif discovery algorithms. However, the model also has inherent limitations that prevent it from accurately representing true binding probabilities, especially for the highest affinity sites under conditions of high protein concentration. The limitations are not due to the assumption of independence between positions but rather are caused by the non-linear relationship between binding affinity and binding probability and the fact that independent normalization at each position skews the site probabilities. Generally probabilistic models are reasonably good approximations, but new high-throughput methods allow for biophysical models with increased accuracy that should be used whenever possible.

## Author summary

Transcription factors (TFs), a class of DNA-binding proteins, play a central role in the regulation of gene expression. TFs control the rate of transcription by binding to the genome in a sequence-specific manner. Thus, one important aspect in the study of gene regulation mechanism is to model the binding specificities of TFs, namely the features of the DNA sequences that a TF prefers to bind. Multiple models have been proposed to characterize the binding specificities of TFs, among which the class of probabilistic models is the most popular. In this study, we point out several major limitations of the well-established probabilistic model by comparing it with the biophysical model. Through simulations we demonstrate that the probabilistic model is only an approximation of the biophysical model. The latter has most of the advantages of the former, and is a more accurate representation of binding specificities. We propose a shift from the probabilistic model to the biophysical model in future studies of protein-DNA interactions.

## Introduction

The study of protein-DNA interactions has a long history and includes binding to both single- and double-stranded DNA and both non-specific and sequence-specific interactions [1]. Our interests are primarily in the sequence-specific interactions of transcription factors (TFs) that bind to DNA to regulate gene expression. Detailed modeling of the *in vivo* interactions of TFs with genomic DNA that control gene expression requires accounting for many complicating factors, including competition and cooperativity with other TFs, competition with nucleosomes for occupancy of specific DNA regions and how sequences flanking the TF binding sites can affect occupancy [2–11]. Regardless of the modeling approach, one component is always the specificity of the TF, how its binding affinity varies depending on the DNA sequence of the binding site. Representations of specificity typically employ matrix-based models where the positions within the binding site are assumed to contribute independently to the TF’s binding affinity [12–14]. In various methods the elements of the matrix may represent probabilities (or log-probabilities) of each base occurring at each position, or energetic contributions from each base at each position, or more generally just abstract scores that are related to the functional contributions of each base at each position [13]. The data used to estimate the matrix parameters may come from many types of either *in vivo* or *in vitro* experiments, employing various types of algorithms [13, 15–38]. To determine the intrinsic specificity of a TF, how its binding affinity varies between different sequences, *in vitro* methods are preferred because the data are unconfounded by the complications that exist *in vivo*. In this paper, we compare two methods of matrix representation of TF specificity, a probabilistic model in which the matrix elements are probabilities of each base at each position, and a biophysical model in which the matrix elements are the binding energy contributions of each base at each position. Either type of matrix could be obtained from the same types of data, and we show that there are inherent limitations of the probabilistic model that do not apply to the biophysical model, suggesting that energy matrices should be preferred in general.

Probabilistic models (*PM*s) for DNA binding proteins were initially introduced by Harr *et al*. for *E*. *coli* promoters that even treated variable length binding sites [39]. Soon after, Staden converted to the use of log-probability to put the model into a weight matrix (additive) model, also including parameters for variable spacing [40]. Schneider *et al*. drew connections between the probabilistic models and information theory and introduced the log-odds model that accounts for the background distribution of bases [41] and later introduced the popular logo graphical representation of specificity [42]. The probabilistic model was also the basis of the earliest motif discovery algorithms [33, 34, 43]. Since then there have been many different algorithms for motif modeling and discovery using probabilistic models (reviewed in [12, 13, 15, 24, 44]).

Even earlier von Hippel introduced an energy-based model of protein-DNA interactions [14]. At the time, there were almost no data on actual binding sites so the paper used first principles to describe the informational specificity required for functional regulatory sites. The paper made simplifying assumptions such as the independence between positions and that every mismatch from the preferred sequence had the same energy difference. The first assumption, of independent contributions, has proven to be a reasonably good approximation for most transcription factors, whereas differences in contributions of alternative bases at each position are now well known and form the basis of most specificity modeling approaches. Berg and von Hippel derived an energy model that was identical to the probabilistic one under some simplifying assumptions and connections between the energy approach and the information theory models of specificity became clear [45–47]. Hwa and colleagues put the energy modeling approach into a more general biophysical model that accounts for the effects of protein concentration on binding probabilities [48, 49]. Djordjevic *et al*. pointed out the importance of the biophysical approach in modeling specificity [50]. They further provided an algorithm that is guaranteed, for any collection of known binding sites, to predict the minimum number of additional sites in a genome, thereby minimizing the number of false positive predictions, although the method is not guaranteed to provide a more accurate model of the true specificity [50, 51]. Regression methods have been used to find optimal energy parameters and Foat *et al*. provided the first regression algorithm for motif discovery of optimal energy models [16, 52]. Since then several related methods have been developed to determine biophysical (energy) models of protein specificity from various types of high-throughput experimental data [18–20, 25, 27, 28, 31, 37, 38, 53–55].

Despite the development of several high-throughput experimental methods for measuring the specificity of protein-DNA interactions [22, 56] and the algorithms described above for modeling them with the biophysical approach, probabilistic models remain the most popular. The purpose of this report is to point out that when good energy models are available there is no advantage to using the probabilistic models. In fact, due to inherent limitations the probabilistic models can be misleading and are highly sensitive to the samples used for inference of the parameters. Energy models can be readily obtained and can easily accommodate non-independent contributions between positions [13, 52, 57]. We conclude that energy modeling should become the approach generally used for modeling specificity and predicting protein-DNA interactions.

## Results

We first introduce the fundamentals of the probabilistic model and the biophysical model and then describe the simulations used to compare the two approaches.

### Probabilistic model

This model is based on a probability matrix *PM*(*b*, *j*) for each base *b* ∈ (*A*, *C*, *G*, *T*) at each position *j* = 1, 2,⋯, *m* for an *m*-long binding site. Any particular DNA sequence *S*_*i*_ can be encoded as a similar matrix, *S*_*i*_(*b*, *j*), of 1s and 0s, where a 1 represents the base that occurs at position *j* and all other elements are 0 [13]. From the model, the probability of the sequence *S*_*i*_ being among the bound sites is:

$$\tilde{P}\left(S_{i}\text{|}B\right)=\prod_{j=1}^{m}\prod_{b=A}^{T}PM{\left(b,j\right)}^{S_{i}\left(b,j\right)}$$ (1)

Often this is converted to a log-odds weight matrix *WM*(*b*, *j*) = log[*PM*(*b*, *j*)/*P*(*b*)], where *P*(*b*) is the background, or prior, probability of base *b* [13, 41]. For simplicity, we assume the prior probability is a constant, 0.25 for each base, and therefore the two approaches give equivalent results. Importantly, this is the probability of observing the sequence *S*_*i*_ given a binding site, whereas what is desired is usually the probability that a sequence *S*_*i*_ is bound, *P*(*B*|*S*_*i*_). Instead, searching sequences with probabilistic models generally just provides a list of sites within some probability range of the preferred site, including a predicted relative probability for each sequence.

### Biophysical model

This model is based on the thermodynamics of the interaction between two molecules, the protein *T* and a binding site *S*_*i*_ (additional details provided in S1 Supporting Information). The association constant, which we refer to as the affinity, can be determined by measuring the concentrations of free reactants (protein and DNA) and of the complex:

$$K_{A}\left(T,S_{i}\right)=\frac{\left[T\cdot S_{i}\right]}{\left[T\right]\left[S_{i}\right]}\equiv K_{i}$$ (2)

It is common to assume that the positions contribute independently to the binding affinity, just as the probabilistic model assumes the positions contribute independently to the site probability. This is represented as a matrix of affinity contributions *K*(*b*, *j*) such that

$$K_{i}=\prod_{j=1}^{m}\prod_{b=A}^{T}K{\left(b,j\right)}^{S_{i}\left(b,j\right)}$$ (3)

From that one can determine the probability of a sequence *S*_*i*_ being bound based on the protein concentration (or really the chemical potential of the protein which is related to its free concentration) and the association constant *K*_*i*_:

$$P\left(B\text{|}S_{i}\right)=\frac{\left[T\cdot S_{i}\right]}{\left[T\cdot S_{i}\right]+\left[S_{i}\right]}=\frac{K_{i}\left[T\right]}{K_{i}\left[T\right]+1}=\frac{1}{1+e^{E_{i}-\mu }}$$ (4)

where *E*_*i*_ = −ln *K*_*i*_ is the free energy of binding to sequence *S*_*i*_ and *μ* = ln[*TF*] is the chemical potential. The probability of sequence *S*_*i*_ in the bound sequences is obtained by Bayes’ rule and is dependent on the chemical potential, which differs from the probabilistic approximation (noted above with $\tilde{P}$):

$$P\left(S_{i}\text{|}B\right)=P(B|S_{i})\frac{P\left(S_{i}\right)}{P\left(B\right)}\propto \frac{K_{i}\left[T\right]}{K_{i}\left[T\right]+1}=\frac{1}{1+e^{E_{i}-\mu }}$$ (5)

if *P*(*S*_*i*_) is the same for all *S*_*i*_. More importantly the true probability of sequence *S*_*i*_ in the bound sequences has a non-linear relationship with its binding affinity. This becomes pronounced at high protein concentrations where the energy can be additive across the positions of the binding site and yet the probabilities of the bases at each position are not independent.

### Simulation results

From Eqs (1) and (5), it is clear that probabilistic models of protein binding specificity provide approximations to true binding probabilities, $\tilde{P}\left(S_{i}\text{|}B\right)\approx P\left(S_{i}\text{|}B\right)$. We used simulations (see the details in the Methods section) to measure the accuracy of the approximation under various values of the chemical potential and for different methods of estimating the *PM* from the observed binding sites. Of particular interest is how well the rank order of binding site probabilities is preserved.

**Fig 1:**
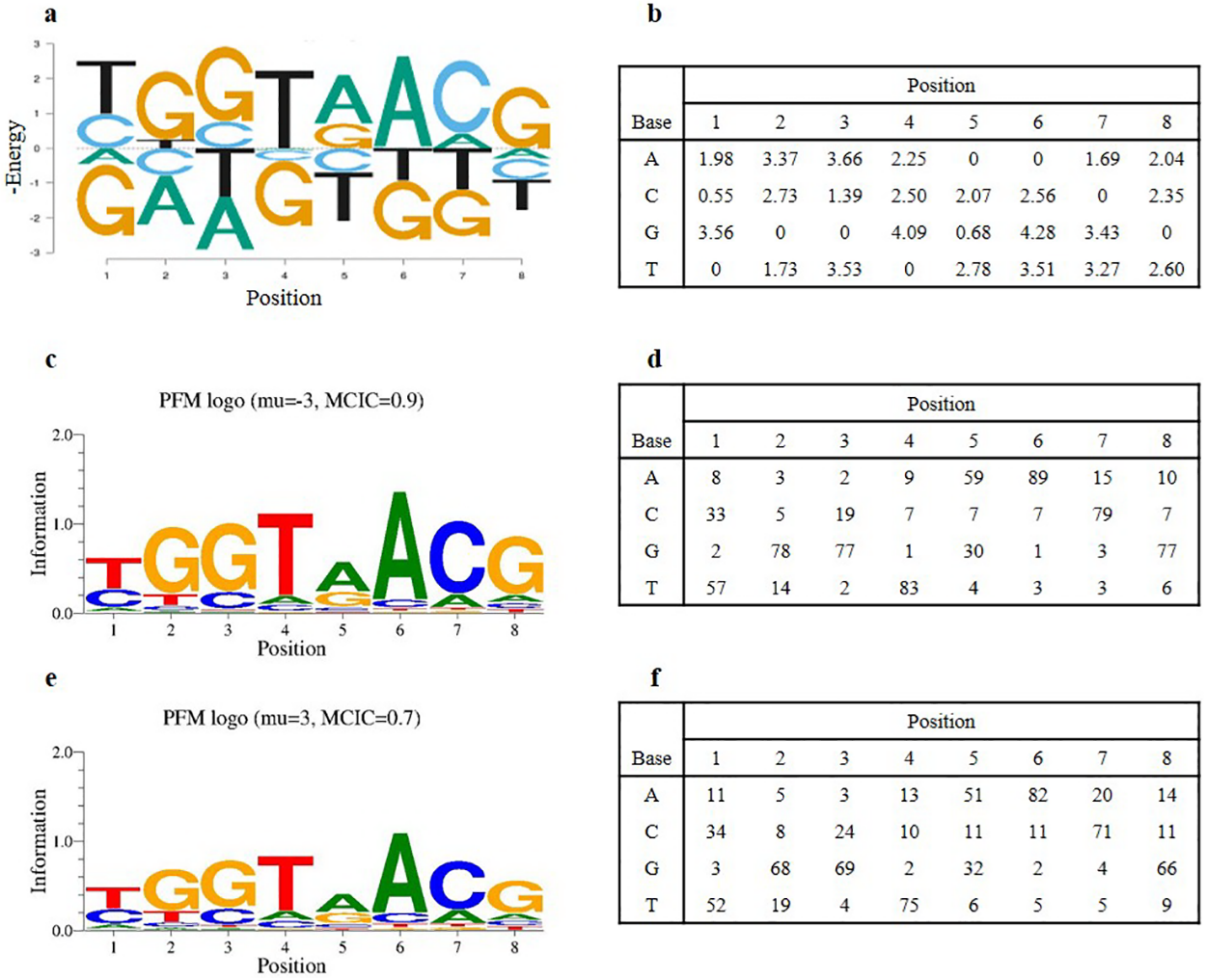
The energy matrix, derived probabilistic models and corresponding logos of a typical simulation. (a) The energy logo, with the average energy for each position set to 0. (b) The energy matrix generated from simulation. (c) The information logo when *μ* = −3. (d) The probabilistic model derived from all binding sites when *μ* = −3. (Matrix elements are frequency of each base at each position; probability × 100.) (e) The information logo when *μ* = 3. (f) The probabilistic model derived from all binding sites when *μ* = 3.

At different protein concentrations, the *PM*s derived from binding probabilities are usually different. As shown in Eq (4), the binding probability of a sequence *S*_*i*_ depends on both its binding affinity *K*_*i*_ (or energy *E*_*i*_) and the protein concentration [*T*] (or chemical potential *μ*). If *K*_*i*_[*T*] ≪ 1, there is a linear relationship between affinity and probability, but that occurs only when *P*(*B*│*S*_*i*_) ≪ 0.5, which is unlikely to be the case *in vivo* for true regulatory sites. At high protein concentrations, where *K*_*i*_[*T*] > 1 and the preferred binding site is highly occupied, the non-linear relationship between binding probability and affinity has several consequences. One is that the *PM* itself depends on the protein concentration, whereas the binding energy does not. Fig 1a and 1b show one example from the simulation of an energy matrix and its associated energy logo [13, 16]. Note that in the matrix the lowest energy base at each position is assigned energy 0 (using the convention of Berg and von Hippel [45]), and in the logo the average energy for each position is set to 0, with the lower energy (higher affinity) bases on top. Since only the differences in energy matter for relative binding affinities, both representations lead to the same results. Fig 1c and 1d show the information logo [42] and the *PM* for that protein obtained at very low protein concentration, *μ* = −3. At low *μ* the *PM* corresponds very closely to the independent contributions of each base to the binding affinity (Eq (5) converges to Eq (1)). But at high protein concentration, such as *μ* = 3, the logo and *PM* are different (Fig 1e and 1f). The second logo shows that the information is “compressed” at *μ* = 3, with the mean column information content (MCIC) decreasing from 0.9 bits to 0.7 bits. But the change in probability is not evenly distributed. Comparing the two *PM*s (Fig 1d and 1f), at high protein concentration the base probabilities tend to move toward the mean, 0.25; the high probability bases decrease in probability and the low probability bases increase. But each position is normalized independently so that the magnitude of the change varies from position to position. For each position, the rank order of probability for the bases remains unchanged, but because the positions are normalized independently, the probabilities of different binding sites may change rank order. That is, even though the binding energy is completely additive across the positions, the probabilities of bases do not factor accurately across the positions.

**Fig 2:**
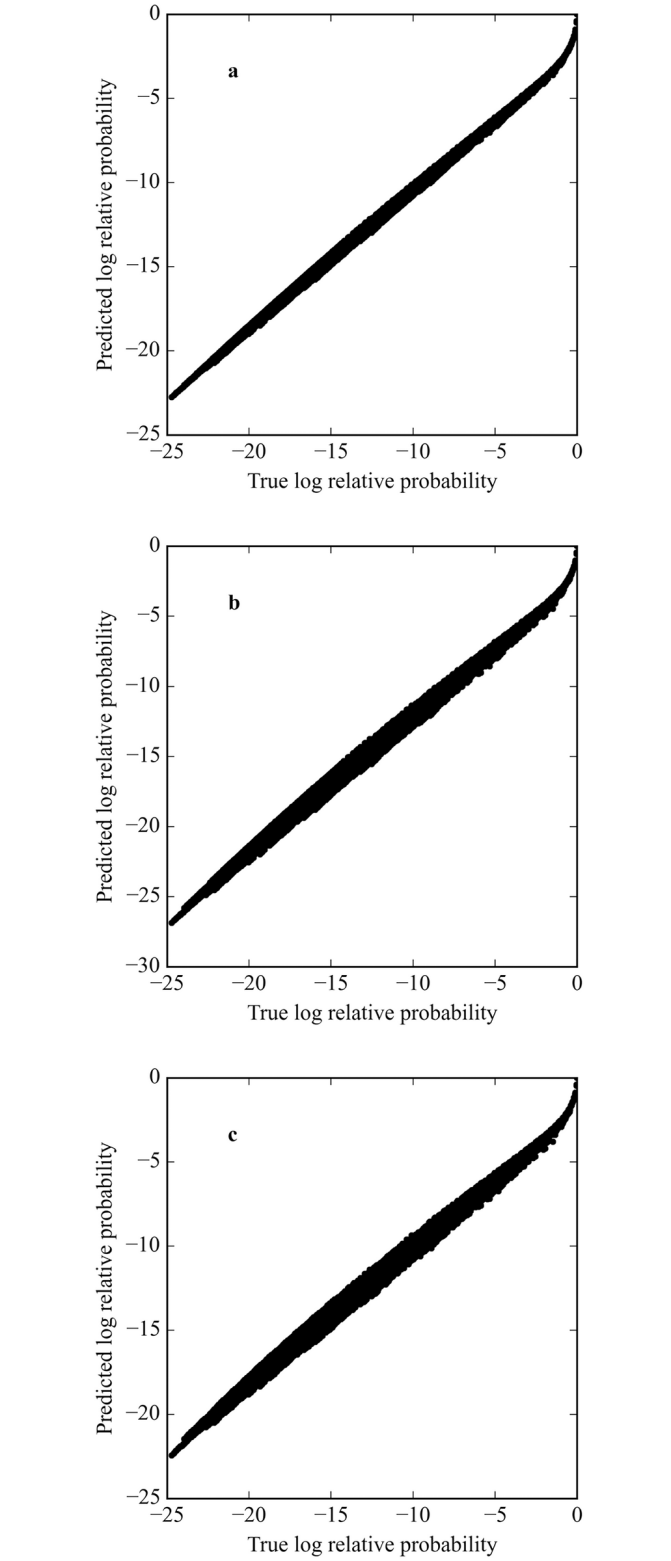
The correlation between the predicted and true all-sequence distributions. (a) The correlation between the true distribution (in logarithm with highest affinity site set to 0) and that predicted by the *PM* generated from the weighted all binding sites. (b) The correlation between the true distribution (in logarithm) and that predicted by the *PM* generated from the weighted top 1% binding sites. (c) The correlation between the true distribution (in logarithm) and that predicted by the *PM* generated from the unweighted top 1% binding sites.

The rank correlation (the square of the Spearman’s rank correlation coefficient) between the predicted and true all-sequence distributions depends on the protein concentration and how the *PM* is computed. Table 1 shows the mean values and standard deviations of *r*^2^ for 100 simulations of 8-long binding sites with *μ* = −3, 0 and 3 (which correspond to the preferred sequence being bound at 0.05, 0.5 and 0.95 probability, respectively). The rank correlation is shown for the complete distribution of binding sites based on *PM*s generated from the full distribution of binding data and from just the top 1% of sites, either weighted or unweighted. At *μ* = −3 there is a nearly perfect fit to the true ranking when the *PM* is derived from the entire distribution. However, when it is based on the top 1% of sites, the ranking is slightly less accurate (0.994) when the sites are weighted by their true probability. In both of those cases the *PM* provides a very good approximation to the true ranking of binding sites. If the top 1% are used unweighted to make the *PM*, the fit to the true ranking is 0.984 and that is true regardless of the value of *μ* because the top 1% of sites is the same and their probabilities are ignored. When *μ* = 0, the results are very similar. For *μ* = 3 the rank correlation drops to 0.988 when the weighted top 1% of sites are used to obtain the *PM*. Fig 2 plots the logarithms of the predicted and true relative binding probabilities for the case of the protein of Fig 1 and *μ* = 3. In each case the overall fit is quite good but the width of the plots indicates some degree of mis-ranking of the binding sites.

**Table 1:** The rank correlation between the predicted and true all sequence distributions.

	Mean correlations and standard deviations		
*PM* generation method	*μ* = −3	*μ* = 0	*μ* = 3
All binding sites, weighted	1.000 (0.000)	1.000 (0.000)	0.998 (0.001)
Top 1% binding sites, weighted	0.994 (0.010)	0.993 (0.011)	0.988 (0.012)
Top 1% binding sites, unweighted	0.984 (0.014)	0.984 (0.014)	0.984 (0.014)

**Fig 3:**
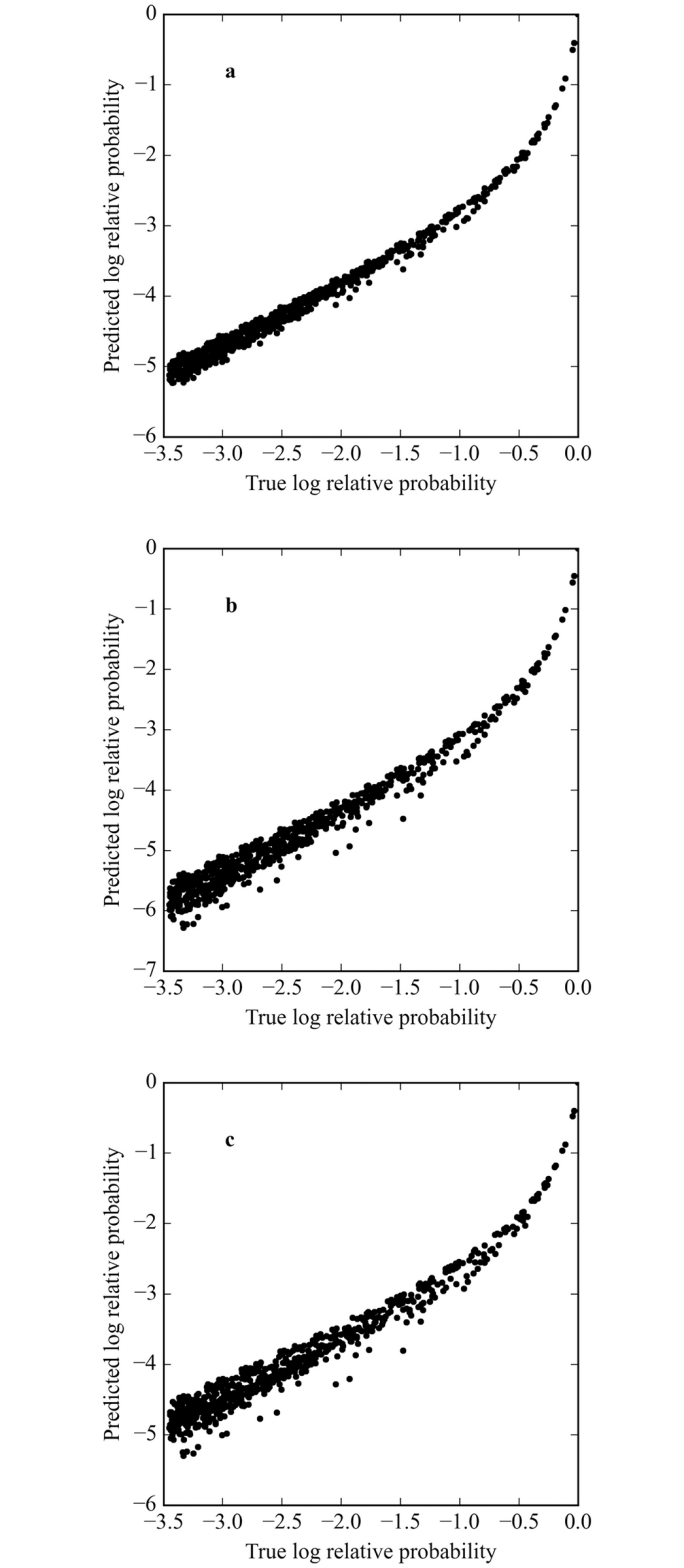
The correlation between the predicted and true top 1% sequence distributions. (a) The correlation between the true distribution (in logarithm with highest affinity site set to 0) and that predicted by the *PM* generated from the weighted all binding sites. (b) The correlation between the true distribution (in logarithm) and that predicted by the *PM* generated from the weighted top 1% binding sites. (c) The correlation between the true distribution (in logarithm) and that predicted by the *PM* generated from the unweighted top 1% binding sites.

While the overall rankings are quite good, it is the highest affinity sites that are of primary interest. In fact, all DNA-binding proteins exhibit a non-specific binding affinity [49] such that there is a minimum binding affinity below which the sequence no longer matters. In addition, functional regulatory sites must have sufficient occupancy to fulfill their roles, so only sites within some restricted range of the optimum are likely to be functional. Table 2 shows the rank correlations for the same *PM*s used in Table 1, but now focusing on the 1% highest affinity binding sites. Note that the values in Table 2 are all lower than for Table 1, indicating that the accuracy is lower when the *PM* is used to predict the highest affinity sites. Fig 3 shows a subset of the plots in Fig 2, including only the top 1% of sites. For *μ* = 3 the rank correlation drops to 0.970 even when the entire distribution is used to generate the *PM* and the scatter of the points shows that there are clear differences between the true and predicted rankings. In fact, the true top 1% is not precisely equivalent to the predicted top 1% of sites. When the top 1% of sites are used to generate the *PM*, weighted by their probability, the rank correlations drop substantially for all values of *μ*, but especially for *μ* = 3 where it is only 0.876. If the unweighted top 1% are used for the *PM*, the rank correlation drops to 0.840 for all values of *μ*. The plots in Fig 3 all show substantial mis-ranking of sites. The results in Tables 1 and 2 show that the quality of *PM*s, their ability to correctly rank binding sites, vary widely depending on both the protein concentration at which the binding data was obtained and the set of binding sites used to derive the *PM*. The effect of the protein concentration is most evident at high values of *μ* where the non-linearity of Eq (4) is largest and non-independence of the position probabilities is most pronounced. The effect of site sampling is due to the sensitivity of the *PM* to the exact set of example sites used.

**Table 2:** The rank correlation between the predicted and true top 1% sequence distributions.

	Mean correlations and standard deviations		
*PM* generation method	*μ* = −3	*μ* = 0	*μ* = 3
All binding sites, weighted	1.000 (0.000)	0.995 (0.002)	0.970 (0.010)
Top 1% binding sites, weighted	0.956 (0.032)	0.930 (0.092)	0.876 (0.063)
Top 1% binding sites, unweighted	0.840 (0.075)	0.840 (0.075)	0.840 (0.075)

**Fig 4:**
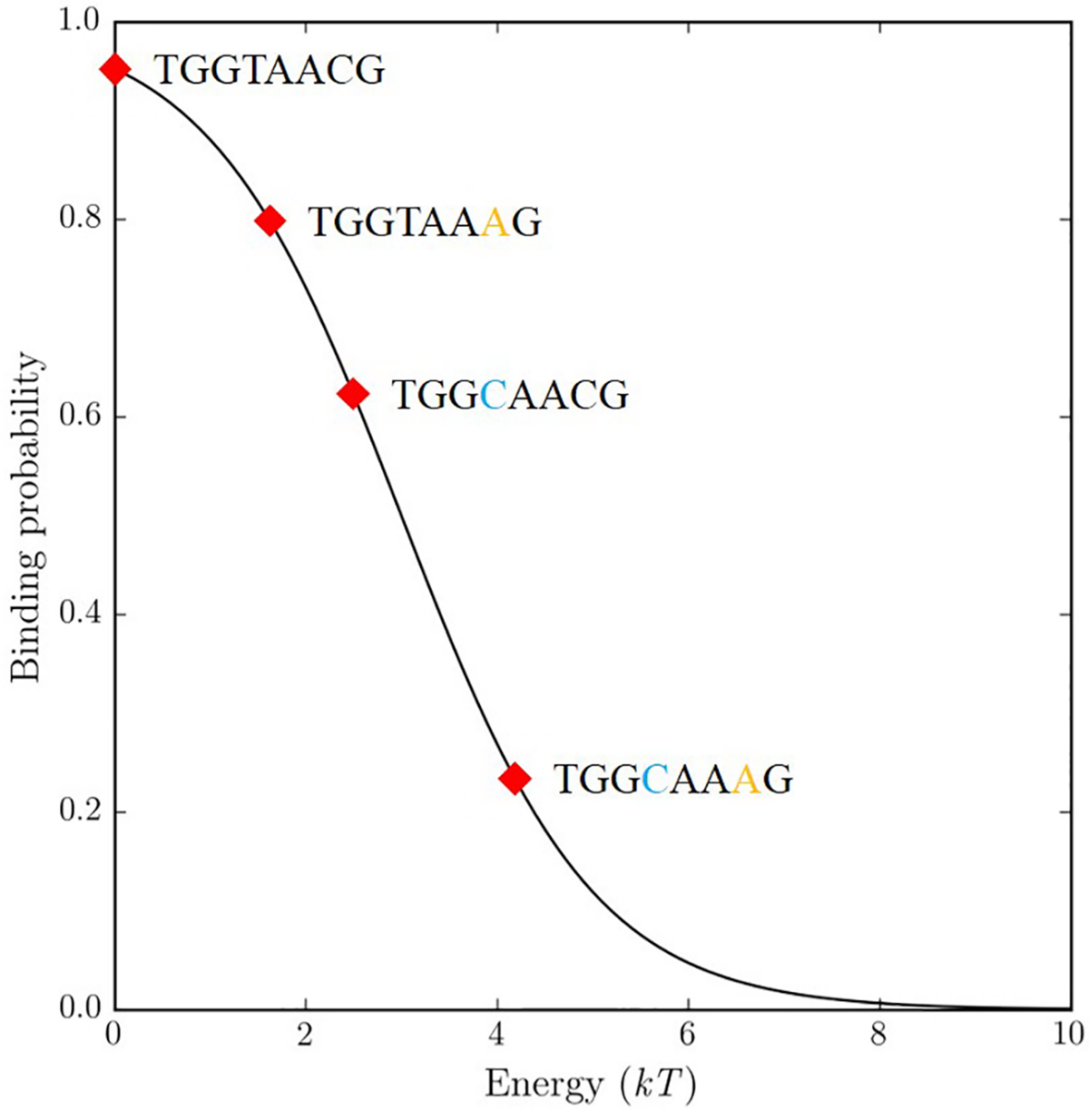
The non-linear relationship between binding affinity and probability. The C to A mutation that occurs at the same position but in two different sequence contexts causes dramatically different changes in the binding probability of the whole sequence.

Another consequence of the non-linear relationship between binding affinity and probability is that pairs (and higher order combinations) of positions have non-independent effects on binding probability, even though the contributions to binding affinity are completely independent. We show this with one example in Fig 4 based on the protein with energy matrix shown in Fig 1 at *μ* = 3. When the preferred binding site, TGGTAACG with binding probability of 0.95, is mutated to TGGTAAAG, the binding probability decreases to 0.79, about a 17% decrease in binding probability. If the same C to A mutation occurs in another sequence, TGG***C***AACG to TGG***C***AAAG, the binding probability decreases from 0.62 to 0.23, a 63% decrease. This apparent non-independence, where the effect of the mutation varies depending its context, is an artifact of the *PM* because the change in binding affinity (1.69 *kT*, Fig 1b) is completely independent of context.

### Effect of noise in experimental data

In the preceding simulations, we compared the probabilistic model to the true biophysical model for the specificity of TFs. However, in real experimental data the observed probabilities of binding to specific sites will include noise that will affect both the probabilistic and biophysical models. To measure the effects of noise on the accuracy of rank predictions, we included errors in the observed probabilities from which both the probabilistic and biophysical matrices were obtained (details in S1 Supporting Information). We added random noise, with mean of 0 and standard deviation of 0.5 *kT*, to the energy of each sequence prior to generating its probability. That amount of noise is larger than what can be obtained with methods such as Spec-seq, where we typically get standard deviationsof about 0.2 *kT*, at least for the high affinity sequences [54, 58]. The results for the probabilistic models are slightly worse than those without noise, described above. For the biophysical models the fits are much better, decreasing to 0.97 when only the top 1% of sites are used to estimate the parameters (see S1 Supporting Information). This is because the noise is added to each sequence independently, whereas the models include the parameters for each base at each position, which are averaged over all of the sequences containing those bases. One advantage of low-dimensional models, such as all types of positional matrices, is that experimental noise is averaged out. In fact, even if the true interaction is not precisely independent between the positions, by averaging over all of the contexts one can obtain models that are good representations of the true specificity.

## Discussion

Probabilistic models of protein-DNA interactions are commonly used because they are easy to obtain and they provide an intuitive representation of specificity. However, they do not provide the information usually desired, the probability that a specific sequence is bound, *P*(*B*│*S*_*i*_), but rather an approximation to the probability of observing a specific sequence given a binding site, $\tilde{P}\left(S_{i}\text{|}B\right)$. From that one can obtain a predicted rank order of all possible binding sites and, if one assumes a specific probability, or occupancy, for the preferred sequence, the predicted probabilities for all other sequences. To obtain binding probabilities from the biophysical model one needs to know the chemical potential, but just as with the probabilistic model if one assumes the probability, or occupancy, of the preferred sequence, then the probabilities of all other sequences can be obtained from the model. Since both models really return the same information, a predicted ranked list of binding sites and relative binding probabilities, they should be judged on the accuracy of those predictions and the ease of obtaining the model parameters.

The accuracy of *PM*s is limited by availability of binding site affinity data. When a *PM* is based on the entire probability distribution of binding sites it is a good approximation overall, even at high *μ*. However, it does have discrepancies that include mis-ordering of the ranks of binding sites as well as the appearance of non-independence between positions that are in fact independent. These effects are due to the intrinsic lack of proportionality between binding probability and binding affinity that is most problematic at high protein concentrations. More severe defects occur due to incomplete information about the binding probability distribution. Obtaining the full distribution of binding probabilities requires *in vitro* experiments, such as protein binding microarrays, HT-SELEX (or SELEX-seq) or other high-throughput methods [20, 22, 27, 35, 53, 59–64], but many algorithms utilize only the highest affinity binding sites. *PM*s can be derived from *in vivo* data of binding site locations, and have the advantage of being easily derived from such data using many motif discovery algorithms [13, 24, 32–34, 43, 65]. But in those cases, the *PM* is derived from only a fraction of the binding sites. Functional regulatory sites will be among the high affinity sequences and in ChIP-seq experiments the peaks will also tend to contain the highest affinity sites. And if the sample size is small, those sites are not even weighted by their binding probabilities. In addition, confounding factors occurring *in vivo*, such as competition and cooperativity with other proteins, lead to incomplete information about the probability distribution and that causes further inaccuracies in the *PM*s.

Good binding models are still important after the advent of high-throughput methods and their parameters can be readily determined by using appropriate algorithms. Binding affinities to small numbers of sequences can be obtained with arbitrarily high accuracy using a variety of experimental techniques [66]. If the additivity (positional independence) assumption is valid, the relative affinities, compared to the preferred sequence, of only the 3*m* single nucleotide variants are needed for the full energy model. Of course, additivity is unlikely to be completely accurate, but there are still only 3*m* + 9(*m* − 1) single variants plus double variants at adjacent positions, where the non-additivity is likely to be most prevalent. But multiple high-throughput methods are now available that provide quantitative binding data from which accurate energy models can be obtained by using appropriate algorithms [16, 18–20, 25, 27, 28, 31, 37, 38, 53, 54, 62]. From sufficiently abundant and accurate quantitative binding data one can even skip the modeling and just use the list of relative binding energies to all possible sites (or at least the highest affinity sites that are likely to function as regulatory sites), avoiding approximations entirely (to the degree allowed by the measurement accuracy). However, models are still useful because they provide a compact representation of specificity, usefully visualized with logos [13, 16, 42], and can provide insight into the mechanisms of binding specificity, such as the contribution of DNA structure to binding specificity [67, 68]. It is also important to have good specificity models obtained from *in vitro* binding experiments to compare to data obtained *in vivo*. This allows one to identify cases where interacting TFs alter the specificity of individual TFs, which one can only infer by having good models for each TF alone [69–71].

We conclude by pointing out that when accurate energy models are available for DNA binding specificity there is no advantage to using probabilistic models, and in fact they can be misleading and provide inaccurate predictions. There are now good high-throughput methods for measuring relative binding affinities to very large collections of sites and good algorithms for determining accurate energy models. We propose that such models become the standard approach for representing specificity and predicting binding sites *in vivo*.

## Methods

### Simulations

We developed a program, BEnDS (Binding Energy Distribution Simulations), to generate random energy matrices of a user-specified length, *m*. One base is randomly chosen as the preferred base at each position and assigned an energy of 0. Energies for the other bases are drawn randomly from a normal distribution with a user-specified mean and standard deviation (with default values of *N*(*μ* = 2.5, *σ* = 1.0)). We assume perfect additivity of binding energies so that the energy for any sequence is the sum of the energies for its bases at each position (equivalent to Eq (3)). This model, implemented in different programs, achieved the best overall performance in a test of various programs on modeling the specificity of DNA-binding proteins based on protein binding microarray (PBM) data [25]. Given an energy matrix, probabilities of binding to all possible sites are obtained using Eq (4) for various (user-specified) values of *μ*. For every set of site probabilities, *PM*s were determined. This was done both for the entire distribution and from a subset of high affinity sites, such as the top 1% (as might be expected to be functional sites). When only the top 1% of sites are used, *PM*s from the sites could be obtained either weighted by their probabilities, or just from the list of sites unweighted, as one might expect from a collection of known regulatory sites or from ChIP-seq type of experiment with a limited sample of observed binding sites.

Simulations that include noise in the energies, and therefore the probabilities, of each sequence are described in S1 Supporting Information. In those cases the energy matrix is obtained using non-linear regression on the site probabilities, similar to BEESEM [37] but without the need to infer the binding site position.

### Measure of rank correlations

The probabilistic model does not attempt to report the probability that a site is bound, *P*(*B*│*S*_*i*_), it only reports the predicted relative probability, and therefore the rank order, of different sites being bound. We compare the true rank order of the sites from their binding energies to the predicted rank order based on the *PM* at different protein concentrations. We report the square of the Spearman’s rank correlation coefficient, *r*^2^.

## Supporting information

S1 Supporting Information Methods for binding probability simulations under measurement noise. (DOCX)

## References

[1] Von Hippel P.H. and McGhee J.D., DNA-protein interactions. Annu Rev Biochem, 1972 41(10): p. 231–300. doi: 10.1146/annurev.bi.41.070172.001311 4570958 10.1146/annurev.bi.41.070172.001311

[2] Granek J.A. and Clarke N.D., Explicit equilibrium modeling of transcription-factor binding and gene regulation. Genome Biol, 2005 6(10): p. R87 doi: 10.1186/gb-2005-6-10-r87 16207358 10.1186/gb-2005-6-10-r87 PMC1257470

[3] Mirny L.A., Nucleosome-mediated cooperativity between transcription factors. Proc Natl Acad Sci U S A, 2010 107(52): p. 22534–9. doi: 10.1073/pnas.0913805107 21149679 10.1073/pnas.0913805107 PMC3012490

[4] Segal E., et al, A genomic code for nucleosome positioning. Nature, 2006 442(7104): p. 772–8. doi: 10.1038/nature04979 16862119 10.1038/nature04979 PMC2623244

[5] Segal E., et al, Predicting expression patterns from regulatory sequence in Drosophila segmentation. Nature, 2008 451(7178): p. 535–40. doi: 10.1038/nature06496 18172436 10.1038/nature06496

[6] Segal E. and Widom J., From DNA sequence to transcriptional behaviour: a quantitative approach. Nat Rev Genet, 2009 10(7): p. 443–56. doi: 10.1038/nrg2591 19506578 10.1038/nrg2591 PMC2719885

[7] Thomas-Chollier M., et al, Transcription factor binding predictions using TRAP for the analysis of ChIP-seq data and regulatory SNPs. Nat Protoc, 2011 6(12): p. 1860–9. doi: 10.1038/nprot.2011.409 22051799 10.1038/nprot.2011.409

[8] Wasson T. and Hartemink A.J., An ensemble model of competitive multi-factor binding of the genome. Genome Res, 2009 19(11): p. 2101–12. doi: 10.1101/gr.093450.109 19720867 10.1101/gr.093450.109 PMC2775586

[9] Afek A., et al, Protein-DNA binding in the absence of specific base-pair recognition. Proc Natl Acad Sci U S A, 2014 111(48): p. 17140–5. doi: 10.1073/pnas.1410569111 25313048 10.1073/pnas.1410569111 PMC4260554

[10] Gordan R., et al, Genomic regions flanking E-box binding sites influence DNA binding specificity of bHLH transcription factors through DNA shape. Cell Rep, 2013 3(4): p. 1093–104. doi: 10.1016/j.celrep.2013.03.014 23562153 10.1016/j.celrep.2013.03.014 PMC3640701

[11] Roider H.G., et al, Predicting transcription factor affinities to DNA from a biophysical model. Bioinformatics, 2007 23(2): p. 134–41. doi: 10.1093/bioinformatics/btl565 17098775 10.1093/bioinformatics/btl565

[12] Stormo G.D., DNA binding sites: representation and discovery. Bioinformatics, 2000 16(1): p. 16–23. 10812473 10.1093/bioinformatics/16.1.16

[13] Stormo G.D., Modeling the specificity of protein-DNA interactions. Quant Biol, 2013 1(2): p. 115–130. doi: 10.1007/s40484-013-0012-4 25045190 10.1007/s40484-013-0012-4 PMC4101922

[14] Von Hippel P.H., On the Molecular Bases of the Specificity of Interaction of Transcriptional Proteins with Genome DNA Biological Regulation and Development. Vol. 1 1979, New York, NY: Plenum Publishing Corp 279–347.

[15] Bussemaker H.J., Foat B.C., and Ward L.D., Predictive modeling of genome-wide mRNA expression: from modules to molecules. Annu Rev Biophys Biomol Struct, 2007 36: p. 329–47. doi: 10.1146/annurev.biophys.36.040306.132725 17311525 10.1146/annurev.biophys.36.040306.132725

[16] Foat B.C., Morozov A.V., and Bussemaker H.J., Statistical mechanical modeling of genome-wide transcription factor occupancy data by MatrixREDUCE. Bioinformatics, 2006 22(14): p. e141–9. doi: 10.1093/bioinformatics/btl223 16873464 10.1093/bioinformatics/btl223

[17] Orenstein Y. and Shamir R., A comparative analysis of transcription factor binding models learned from PBM, HT-SELEX and ChIP data. Nucleic Acids Res, 2014 42(8): p. e63 doi: 10.1093/nar/gku117 24500199 10.1093/nar/gku117 PMC4005680

[18] Orenstein Y. and Shamir R. HTS-IBIS: fast and accurate inference of binding site motifs from HT-SELEX data. bioRxiv, 2015 doi: 10.1101/022277

[19] Riley T.R., et al, Building accurate sequence-to-affinity models from high-throughput in vitro protein-DNA binding data using FeatureREDUCE. Elife, 2015 4. 10.7554/eLife.06397 PMC4758951 26701911

[20] Riley T.R., et al, SELEX-seq: a method for characterizing the complete repertoire of binding site preferences for transcription factor complexes. Methods Mol Biol, 2014 1196: p. 255–78. doi: 10.1007/978-1-4939-1242-1_16 25151169 10.1007/978-1-4939-1242-1_16 PMC4265583

[21] Roulet E., et al, High-throughput SELEX SAGE method for quantitative modeling of transcription-factor binding sites. Nat Biotechnol, 2002 20(8): p. 831–5. doi: 10.1038/nbt718 12101405 10.1038/nbt718

[22] Stormo G.D. and Zhao Y., Determining the specificity of protein-DNA interactions. Nat Rev Genet, 2010 11(11): p. 751–60. doi: 10.1038/nrg2845 20877328 10.1038/nrg2845

[23] Stormo G.D., Zuo Z., and Chang Y.K., Spec-seq: determining protein-DNA-binding specificity by sequencing. Brief Funct Genomics, 2015 14(1): p. 30–8. doi: 10.1093/bfgp/elu043 25362070 10.1093/bfgp/elu043 PMC4366588

[24] van Nimwegen E., Finding regulatory elements and regulatory motifs: a general probabilistic framework. BMC Bioinformatics, 2007 8 Suppl 6: p. S4. 10.1186/1471-2105-8-S6-S4 PMC1995539 17903285

[25] Weirauch M.T., et al, Evaluation of methods for modeling transcription factor sequence specificity. Nat Biotechnol, 2013 31(2): p. 126–34. doi: 10.1038/nbt.2486 23354101 10.1038/nbt.2486 PMC3687085

[26] Weirauch M.T., et al, Determination and inference of eukaryotic transcription factor sequence specificity. Cell, 2014 158(6): p. 1431–43. doi: 10.1016/j.cell.2014.08.009 25215497 10.1016/j.cell.2014.08.009 PMC4163041

[27] Zhao Y., Granas D., and Stormo G.D., Inferring binding energies from selected binding sites. PLoS Comput Biol, 2009 5(12): p. e1000590 doi: 10.1371/journal.pcbi.1000590 19997485 10.1371/journal.pcbi.1000590 PMC2777355

[28] Zhao Y. and Stormo G.D., Quantitative analysis demonstrates most transcription factors require only simple models of specificity. Nat Biotechnol, 2011 29(6): p. 480–3. 10.1038/nbt.1893 PMC3111930 21654662

[29] Liu X., Brutlag D.L., and Liu J.S., BioProspector: discovering conserved DNA motifs in upstream regulatory regions of co-expressed genes. Pac Symp Biocomput, 2001: p. 127–38. 11262934

[30] Liu X., et al, DIP-chip: rapid and accurate determination of DNA-binding specificity. Genome Res, 2005 15(3): p. 421–7. doi: 10.1101/gr.3256505 15710749 10.1101/gr.3256505 PMC551568

[31] Locke G. and Morozov A.V., A Biophysical Approach to Predicting Protein-DNA Binding Energetics. Genetics, 2015 200(4): p. 1349–61. doi: 10.1534/genetics.115.178384 26081193 10.1534/genetics.115.178384 PMC4574261

[32] Hertz G.Z. and Stormo G.D., Identifying DNA and protein patterns with statistically significant alignments of multiple sequences. Bioinformatics, 1999 15(7–8): p. 563–77. 10487864 10.1093/bioinformatics/15.7.563

[33] Lawrence C.E., et al, Detecting subtle sequence signals: a Gibbs sampling strategy for multiple alignment. Science, 1993 262(5131): p. 208–14. 8211139 10.1126/science.8211139

[34] Lawrence C.E. and Reilly A.A., An expectation maximization (EM) algorithm for the identification and characterization of common sites in unaligned biopolymer sequences. Proteins, 1990 7(1): p. 41–51. doi: 10.1002/prot.340070105 2184437 10.1002/prot.340070105

[35] Jolma A., et al, Multiplexed massively parallel SELEX for characterization of human transcription factor binding specificities. Genome Res, 2010 20(6): p. 861–73. doi: 10.1101/gr.100552.109 20378718 10.1101/gr.100552.109 PMC2877582

[36] Jolma A., et al, DNA-binding specificities of human transcription factors. Cell, 2013 152(1–2): p. 327–39. doi: 10.1016/j.cell.2012.12.009 23332764 10.1016/j.cell.2012.12.009

[37] Ruan S., Joshua Swamidass S., and Stormo G.D., BEESEM: Estimation of Binding Energy Models Using HT-SELEX Data. Bioinformatics, 2017. 10.1093/bioinformatics/btx191 PMC5860122 28379348

[38] Atherton J., et al, *A model for sequential evolution of ligands by exponential enrichment (SELEX) data*. 2012: p. 928–949.

[39] Harr R., Haggstrom M., and Gustafsson P., Search algorithm for pattern match analysis of nucleic acid sequences. Nucleic Acids Res, 1983 11(9): p. 2943–57. 6344023 10.1093/nar/11.9.2943 PMC325935

[40] Staden R., Computer methods to locate signals in nucleic acid sequences. Nucleic Acids Res, 1984 12(1 Pt 2): p. 505–19. 6364039 10.1093/nar/12.1part2.505 PMC321067

[41] Schneider T.D., et al, Information content of binding sites on nucleotide sequences. J Mol Biol, 1986 188(3): p. 415–31. 3525846 10.1016/0022-2836(86)90165-8

[42] Schneider T.D. and Stephens R.M., Sequence logos: a new way to display consensus sequences. Nucleic Acids Res, 1990 18(20): p. 6097–100. 2172928 10.1093/nar/18.20.6097 PMC332411

[43] Stormo G.D. and Hartzell G.W. 3rd, Identifying protein-binding sites from unaligned DNA fragments. Proc Natl Acad Sci U S A, 1989 86(4): p. 1183–7. 2919167 10.1073/pnas.86.4.1183 PMC286650

[44] D'Haeseleer P., How does DNA sequence motif discovery work? Nat Biotechnol, 2006 24(8): p. 959–61. doi: 10.1038/nbt0806-959 16900144 10.1038/nbt0806-959

[45] Berg O.G. and von Hippel P.H., Selection of DNA binding sites by regulatory proteins. Statistical-mechanical theory and application to operators and promoters. J Mol Biol, 1987 193(4): p. 723–50. 3612791 10.1016/0022-2836(87)90354-8

[46] Stormo G.D., Computer methods for analyzing sequence recognition of nucleic acids. Annu Rev Biophys Biophys Chem, 1988 17: p. 241–63. doi: 10.1146/annurev.bb.17.060188.001325 3293587 10.1146/annurev.bb.17.060188.001325

[47] Stormo G.D. and Fields D.S., Specificity, free energy and information content in protein-DNA interactions. Trends Biochem Sci, 1998 23(3): p. 109–13. 9581503 10.1016/s0968-0004(98)01187-6

[48] Bintu L., et al, Transcriptional regulation by the numbers: models. Curr Opin Genet Dev, 2005 15(2): p. 116–24. doi: 10.1016/j.gde.2005.02.007 15797194 10.1016/j.gde.2005.02.007 PMC3482385

[49] Gerland U., Moroz J.D., and Hwa T., Physical constraints and functional characteristics of transcription factor-DNA interaction. Proc Natl Acad Sci U S A, 2002 99(19): p. 12015–20. doi: 10.1073/pnas.192693599 12218191 10.1073/pnas.192693599 PMC129390

[50] Djordjevic M., Sengupta A.M., and Shraiman B.I., A biophysical approach to transcription factor binding site discovery. Genome Res, 2003 13(11): p. 2381–90. doi: 10.1101/gr.1271603 14597652 10.1101/gr.1271603 PMC403756

[51] Homsi D.S., Gupta V., and Stormo G.D., Modeling the quantitative specificity of DNA-binding proteins from example binding sites. PLoS One, 2009 4(8): p. e6736 doi: 10.1371/journal.pone.0006736 19707584 10.1371/journal.pone.0006736 PMC2726951

[52] Stormo G.D., Schneider T.D., and Gold L., Quantitative analysis of the relationship between nucleotide sequence and functional activity. Nucleic Acids Res, 1986 14(16): p. 6661–79. 3092188 10.1093/nar/14.16.6661 PMC311672

[53] Fordyce P.M., et al, De novo identification and biophysical characterization of transcription-factor binding sites with microfluidic affinity analysis. Nat Biotechnol, 2010 28(9): p. 970–5. doi: 10.1038/nbt.1675 20802496 10.1038/nbt.1675 PMC2937095

[54] Zuo Z. and Stormo G.D., High-resolution specificity from DNA sequencing highlights alternative modes of Lac repressor binding. Genetics, 2014 198(3): p. 1329–43. doi: 10.1534/genetics.114.170100 25209146 10.1534/genetics.114.170100 PMC4224169

[55] Liu J. and Stormo G.D., Combining SELEX with quantitative assays to rapidly obtain accurate models of protein-DNA interactions. Nucleic Acids Res, 2005 33(17): p. e141 doi: 10.1093/nar/gni139 16186128 10.1093/nar/gni139 PMC1236725

[56] Bussemaker H.J., Recent progress in understanding transcription factor binding specificity. Brief Funct Genomics, 2015 14(1): p. 1–2. doi: 10.1093/bfgp/elu050 25617355 10.1093/bfgp/elu050

[57] Zhao Y., et al, Improved models for transcription factor binding site identification using nonindependent interactions. Genetics, 2012 191(3): p. 781–90. doi: 10.1534/genetics.112.138685 22505627 10.1534/genetics.112.138685 PMC3389974

[58] Roy B., Zuo Z., and Stormo G.D., Quantitative specificity of STAT1 and several variants. Nucleic Acids Res, 2017. 10.1093/nar/gkx393 PMC5737217 28510715

[59] Berger M.F., et al, Compact, universal DNA microarrays to comprehensively determine transcription-factor binding site specificities. Nat Biotechnol, 2006 24(11): p. 1429–35. doi: 10.1038/nbt1246 16998473 10.1038/nbt1246 PMC4419707

[60] Orenstein Y. and Shamir R., A comparative analysis of transcription factor binding models learned from PBM, HT-SELEX and ChIP data. Nucleic Acids Research, 2014 42(8). 10.1093/nar/gku117 PMC4005680 24500199

[61] Zykovich A., Korf I., and Segal D.J., Bind-n-Seq: high-throughput analysis of in vitro protein-DNA interactions using massively parallel sequencing. Nucleic Acids Res, 2009 37(22): p. e151 doi: 10.1093/nar/gkp802 19843614 10.1093/nar/gkp802 PMC2794170

[62] Isakova A., et al, SMiLE-seq identifies binding motifs of single and dimeric transcription factors. Nat Methods, 2017 14(3): p. 316–322. doi: 10.1038/nmeth.4143 28092692 10.1038/nmeth.4143

[63] Maerkl S.J. and Quake S.R., A systems approach to measuring the binding energy landscapes of transcription factors. Science, 2007 315(5809): p. 233–7. doi: 10.1126/science.1131007 17218526 10.1126/science.1131007

[64] Nutiu R., et al, Direct measurement of DNA affinity landscapes on a high-throughput sequencing instrument. Nat Biotechnol, 2011 29(7): p. 659–64. doi: 10.1038/nbt.1882 21706015 10.1038/nbt.1882 PMC3134637

[65] Bailey T.L., et al, The MEME Suite. Nucleic Acids Res, 2015 43(W1): p. W39–49. doi: 10.1093/nar/gkv416 25953851 10.1093/nar/gkv416 PMC4489269

[66] Stormo G., *Introduction to protein-DNA interactions: structure, thermodynamics, and bioinformatics*. 2013, Cold Spring Harbor, N.Y.: Cold Spring Harbor Laboratory Press x, 198 p.

[67] Mathelier A., et al, DNA Shape Features Improve Transcription Factor Binding Site Predictions In Vivo. Cell Syst, 2016 3(3): p. 278–286 e4. doi: 10.1016/j.cels.2016.07.001 27546793 10.1016/j.cels.2016.07.001 PMC5042832

[68] Yang L., et al, Transcription factor family-specific DNA shape readout revealed by quantitative specificity models. Mol Syst Biol, 2017 13(2): p. 910 doi: 10.15252/msb.20167238 28167566 10.15252/msb.20167238 PMC5327724

[69] Jolma A., et al, DNA-dependent formation of transcription factor pairs alters their binding specificity. Nature, 2015 527(7578): p. 384–8. doi: 10.1038/nature15518 26550823 10.1038/nature15518

[70] Slattery M., et al, Cofactor binding evokes latent differences in DNA binding specificity between Hox proteins. Cell, 2011 147(6): p. 1270–82. doi: 10.1016/j.cell.2011.10.053 22153072 10.1016/j.cell.2011.10.053 PMC3319069

[71] Slattery M., et al, Absence of a simple code: how transcription factors read the genome. Trends Biochem Sci, 2014 39(9): p. 381–99. doi: 10.1016/j.tibs.2014.07.002 25129887 10.1016/j.tibs.2014.07.002 PMC4149858

