# Supplementary material for: Inherent limitations of probabilistic models for protein-DNA binding specificity
Source: PLoS Comput Biol. 2017 Jul 7;13(7):e1005638. doi: 10.1371/journal.pcbi.1005638 (PMC5521849; doi:10.1371/journal.pcbi.1005638)
Supplement: S1 Supporting Information — (DOCX) [file pcbi.1005638.s001.docx]

Supplementary Information

Methods for Binding Probability Simulations Under Measurement Noise

**The Biophysical Model of Binding Specificity**

The biophysical model is the same as the one described in the main text. Based on equation (4), we can compute the probability of a sequence $S_{i}$ being bound:

$P\left( B | S_{i} \right)=\frac{1}{1+e^{E_{i}-\mu}}$ (S1)

In the above equation, the total binding energy $E_{i}$ for the sequence $S_{i}$ is calculated using the following equation:

$E_{i}=\sum_{j=1}^{m} \sum_{b=A}^{T} E\left( b,j \right)S_{i}\left( b,j \right)$ (S2)

$E\left( b,j \right)$ is a 4-by-*m* energy matrix, where *m* is the motif length. $S_{i}\left( b,j \right)$ is the encoding of sequence $S_{i}$, as described in the main text. Equation (S2) assumes that the positions contribute independently to the total binding energy. $E\left( b,j \right)$ is related to the binding affinity (defined in equation (2) and (3)) through the following equation:

$E\left( b,j \right)=-\ln K\left( b,j \right)$ (S3)

According to equation (5), if we assume an equiprobable prior distribution for all the sequences, we can compute $P\left( S_{i} | B \right)$, the probability of sequence $S_{i}$ among the bound sequences, by simply normalizing $P\left( B | S_{i} \right)$.

**The True Parameters for the Biophysical Model**

To generate a set of true parameters for the biophysical model, the user needs to specify the motif length *m* and the chemical potential *µ*. Then we use a program to randomly generate a 4-by-*m* energy matrix $E\left( b,j \right)$. The chemical potential *µ* and the generated energy matrix $E\left( b,j \right)$ will serve as the true parameters. To generate binding probabilities, we first compute the binding energy of sequence $S_{i}$ using equation (S2). Then we compute $P\left( B | S_{i} \right)$ using equation (S1). By normalizing $P\left( B | S_{i} \right)$ we obtain the clean probability of sequence $S_{i}$ among the bound sequences, denoted by $P\left( S_{i} | B \right)$. The clean probability $P\left( S_{i} | B \right)$ is mainly used to evaluate the predictions of the fitted biophysical models and the derived probabilistic models (described in following sections).

To add measurement noise, we randomly generate an error $\epsilon_{i}$ from the normal distribution $N\left( \mu=0, \sigma=0.5 \right)$. Then we calculate the binding energy of sequence $S_{i}$ under noise:

$E_{i}^{*}=E_{i}+\epsilon_{i}$ (S4)

Then we use $E_{i}^{*}$ to compute the probability of a sequence $S_{i}$ being bound under noise:

$P^{*}\left( B | S_{i} \right)=\frac{1}{1+e^{E_{i}^{*}-\mu}}$ (S5)

Finally, we normalize $P^{*}\left( B | S_{i} \right)$ to obtain the noisy probability $P^{*}\left( S_{i} | B \right)$, which is the observed probability of sequence $S_{i}$ among the bound sequences. $P^{*}\left( S_{i} | B \right)$ is used to estimate the parameters in the fitted biophysical model and the probabilistic models.

**The Fitted Biophysical Model**

To fit a biophysical model to the noisy observation $P^{*}\left( S_{i} | B \right)$, we solve the following optimization problem:

$\min_{A, \mu, E\left( b,j \right)} \sum_{i} \left[ \ln\frac{A}{1+e^{E_{i}-\mu}}-\ln P^{*}\left( S_{i} | B \right) \right]^{2}$ (S6)

In the above equation, $E_{i}$ is computed using equation (S2). The unknown parameters that needs to be estimated are the scale factor *A*, the chemical potential *µ* and the energy matrix $E\left( b,j \right)$. The optimization problem is solved using the L-BFGS-B algorithm. With the estimated parameters, we can compute the $\hat{P}\left( S_{i} | B \right)$ predicted by the fitted biophysical model and compare it against the clean probability $P\left( S_{i} | B \right)$. We will generate two types of fitted biophysical models. They are estimated using either all the sequences or the top 1% sequences ranked by $P^{*}\left( S_{i} | B \right)$.

**The Estimated Probabilistic Models**

The noisy observation $P^{*}\left( S_{i} | B \right)$ is used to compute the probabilistic models (PMs) using the same procedure as described in the main text. This was done both for the entire distribution and from a subset of high affinity sites, such as the top 1% (as might be expected to be functional sites). When only the top 1% of sites are used, PMs from the sites could be obtained either weighted by their probabilities, or just from the list of sites unweighted.

**The Rank Correlation Between the Predicted and True Sequence Distributions**

To evaluate the accuracy of the fitted biophysical models and the estimated PMs, we computed the rank correlation between their predicted all sequence or top 1% sequence distributions $\hat{P}\left( S_{i} | B \right)$ and the true distribution (namely the clean probability $P\left( S_{i} | B \right)$). The results are summarized in the tables below. Three main conclusions can be drawn from the results. First, the fitted biophysical models can recover the true parameters used to generate the noisy observation $P^{*}\left( S_{i} | B \right)$, as indicated by the good correlation between the predicted and true all-sequence or top 1% sequence distributions (Table S3 and Table S4). Second, comparison of Table 1 and Table S1 shows that when all the sequences are considered, the overall accuracy of the PMs derived from the noisy observation $P^{*}\left( S_{i} | B \right)$ is essentially the same as those derived from the clean observation $P\left( S_{i} | B \right)$ (described in the main text), as their average rank correlation scores are very similar. Finally, comparison of Table 2 and Table S2 shows that when only the top 1% sequences are considered, the accuracy of the PMs derived from the noisy observation $P^{*}\left( S_{i} | B \right)$ is generally lower than those derived from the clean observation $P\left( S_{i} | B \right)$, especially when $\mu=-3$ or $\mu=0$.

Table S1. Rank correlation between predicted and true all-sequence distributions for probabilistic models.

|  | Mean correlations and standard deviations | | |
| --- | --- | --- | --- |
| PM generation method | $\mu=-3$ | $\mu=0$ | $\mu=3$ |
| All binding sites, weighted | 0.997 (0.002) | 0.997 (0.001) | 0.997 (0.001) |
| Top 1% binding sites, weighted | 0.987 (0.030) | 0.988 (0.019) | 0.987 (0.022) |
| Top 1% binding sites, unweighted | 0.983 (0.014) | 0.984 (0.015) | 0.986 (0.008) |

Table S2. Rank correlation between predicted and true top 1% sequence distributions for probabilistic models.

|  | Mean correlations and standard deviations | | |
| --- | --- | --- | --- |
| PM generation method | $\mu=-3$ | $\mu=0$ | $\mu=3$ |
| All binding sites, weighted | 0.952 (0.022) | 0.959 (0.016) | 0.959 (0.013) |
| Top 1% binding sites, weighted | 0.886 (0.090) | 0.883 (0.035) | 0.872 (0.043) |
| Top 1% binding sites, unweighted | 0.839 (0.069) | 0.842 (0.066) | 0.846 (0.052) |

Table S3. Rank correlation between predicted and true all-sequence distributions for biophysical models.

|  | Mean correlations and standard deviations | | |
| --- | --- | --- | --- |
| Biophysical model generation method | $\mu=-3$ | $\mu=0$ | $\mu=3$ |
| All binding sites, weighted | 1.000 (0.000) | 1.000 (0.000) | 1.000 (0.000) |
| Top 1% binding sites, weighted | 0.992 (0.034) | 0.995 (0.022) | 0.995 (0.023) |

Table S4. Rank correlation between predicted and true top 1% sequence distributions for biophysical models.

|  | Mean correlations and standard deviations | | |
| --- | --- | --- | --- |
| Biophysical model generation method | $\mu=-3$ | $\mu=0$ | $\mu=3$ |
| All binding sites, weighted | 1.000 (0.000) | 1.000 (0.000) | 1.000 (0.000) |
| Top 1% binding sites, weighted | 0.964 (0.092) | 0.972 (0.009) | 0.968 (0.012) |
